# The limited capacity of visual temporal integration in cats

**DOI:** 10.1167/jov.20.8.28

**Published:** 2020-08-27

**Authors:** Xiaohan Bao, Anas Salloum, Stephen G. Gordon, Stephen G. Lomber

**Affiliations:** Integrated Program in Neuroscience, McGill University, Montreal, Quebec, Canada; Department of Physiology, Faculty of Medicine, McGill University, Montreal, Quebec, Canada; Undergraduate Program in Physiology and Pharmacology, University of Western Ontario, London, Ontario, Canada

**Keywords:** temporal integration, cat visual psychophysics, Bloch's law

## Abstract

It has been long known that prolonging stimulus duration may increase the perceived brightness of a visual stimulus. The interaction between intensity and duration generally follows a rule, such as that described in Bloch's law. This visual temporal integration relationship has been identified in human subjects and in non-human primates. However, although auditory temporal integration has been extensively studied in the cat, visual temporal integration has not. Therefore, the goal of this study was to examine visual temporal integration in the cat. We trained five cats to respond when a brief luminance change was detected in a fixation dot. After training, we measured the success rate of detecting the luminance change with varying durations at threshold, subthreshold, and suprathreshold luminance levels. Psychometric functions showed that prolonging stimulus duration improved task performance, more noticeably for stimuli below 100 ms than beyond. Most psychometric functions were better fit to an exponential model than to a linear model. The gradually saturated performance observed here, as in previous studies, can be explained by the “leaky integrator” hypothesis, that is, temporal integration is only valid below a critical duration. Overall, we developed a task whereby visual temporal integration was successfully demonstrated in the cat. The effect of stimulus duration on detection success rate displayed a pattern generally consistent with previous human and non-human primate findings on visual temporal integration.

## Introduction

The temporal resolution of a sensory system is limited by the neurobiological basis of the receptor organ and the central nervous system. This nature can be reflected as a perceptual ambiguity between the duration and intensity of a stimulus. For example, the human vision system, in some circumstances, interprets the prolongation of stimulus duration as an increase in brightness, whereas stimulus luminance remains the same. Since it was initially documented 135 years ago (Bloch, 1885), this phenomenon has been extensively examined (Breitmeyer & Ganz, 1977; Gorea & Tyler, 1986; Kelly & Savoie, 1978; Rieiro et al., 2012; Roufs, 1974). In these studies, this phenomenon was often illustrated by iso-brightness curves in a stimulus luminance–duration domain, where a stimulus with a long duration and low luminance has the same perceived brightness as another stimulus with a short duration and high luminance. Such a trade-off between luminance and duration shows that the visual system can integrate stimulus input over time to boost stimulus intensity at the expense of temporal resolution.

Meanwhile, because this trade-off is quantified with psychometric functions, many studies have identified that the trade-off ratio varies with stimulus duration. For example, Aiba and Stevens (1964) found that the effect of doubling the stimulus duration on perceived brightness was equivalent to increasing luminance 10 times (decoupling stimulus luminance) for stimuli shorter than 10 ms, but became noticeably smaller for stimuli 10 to 100 ms long, and eventually could not be detected for stimuli longer than 100 ms. The marginal perceptual benefit of stimulus duration approaching zero, thus marking the “saturation” of the temporal integration window, is also known as the “critical duration.” A few studies have reported a small decrease in the perceived brightness for medium–long duration stimuli (Gorea & Tyler, 1986; Kelly & Savoie, 1978; Rieiro et al., 2012). However, it is still being debated whether this phenomenon is due to a participant bias (Rieiro et al., 2012) or lateral inhibition (Gorea & Tyler, 1986, 2013).

A number of studies have examined the neural mechanism of visual temporal integration (Duysens, Gulyás, & Maes, 1991; Goris, Ziemba, Movshon, & Simoncelli, 2018; Levick & Zacks, 1970; Ohtani, Okamura, & Ejima, 2002; Scheich & Korn, 1971; White & Jeffreys, 1982). For example, it has been recently shown that an integrating neuron coding stimulus intensity by means of firing rate can be modulated by stimulus duration below a “neuronal” critical duration (Goris et al., 2018). Beyond that critical duration, such modulation became weaker and eventually absent.

Although these studies have shed some light on the neural correlates of temporal integration, future studies are still needed to explore its modality specificity and significance to behavior. To achieve these goals, it will be advantageous to have an animal model that is convenient for both electrophysiological and behavioral studies in both the auditory and visual systems. It has already been shown that cat area 17 neurons demonstrate features of temporal integration (Duysens et al., 1991). However, to the best of our knowledge, no prior study has psychophysically examined visual temporal integration in the cat.

Overall, in this investigation we developed a visual task for the cat to establish a new animal model for studying temporal integration. The effect of stimulus duration on the success rate of detection was examined and displayed a pattern generally consistent with previous findings of visual temporal integration in humans and non-human primates.

## Methods

All procedures were conducted in compliance with the National Research Council's Guide for the Care and Use of Laboratory Animals (8th edition; 2011) and the Canadian Council on Animal Care's Guide to the Care and Use of Experimental Animals (1993), and adhere to the ARVO Animal Statement. Furthermore, the following procedures were also approved by the University of Western Ontario's Animal Use Subcommittee of the University Council on Animal Care.

### Subjects

All subjects were domestic short hair cats derived from a commercial breeding facility (Marshall BioResources, formerly Liberty Laboratories, Waverly, NY). The animals are group housed with other cats in an enriched environment with a 12-hour light cycle and ad libitum water. Their health was monitored by a veterinary technician on a daily basis and by a veterinarian once per week. The facility was regularly inspected by the Ontario Ministry of Agriculture, Food, and Rural Affairs, and the Canadian Council on Animal Care.

Five cats (TRN, CHB, CTL, ARY, and BRN) were trained and tested in this experiment; most of them are also participating in other projects. Concerning the other projects, TRN was surgically implanted with a multichannel recording microelectrode in its left primary auditory cortex 2 months before testing for this experiment. ARY and BRN were deafened via daily subcutaneous injections of neomycin (60 mg/kg) for 30 days after birth (Leake, Hradek, Rebscher, & Snyder, 1991). No signs of weight loss or abnormal behavior except for the loss of hearing in ARY and BRN was noticed. Food was provided ad libitum for 1 hour per day after their daily training or testing.

### Equipment and software

Before training, the cats were conditioned to be loosely restrained by a canvas bag that was attached to a cat chair, on which the cat could comfortably stand or sit while keeping their heads in a limited region facing toward a monitor. The monitor (XL2820, BenQ) was 59.8 cm wide × 33.6 cm high with a resolution of 1920 × 1080 and a refresh rate of 60 Hz and was placed horizontally approximately 40 cm in front of the cat. The visual stimulus was programmed and rendered in PsychToolbox on Matlab utilizing PCI graphic cards (Graphics Chipset AMD Radeon R9 200 Series).

Only positive reinforcement was used. Moist canned food was delivered with an autofilled spoon placed between the cat and the monitor in such a way as to not block the cat's view of the monitor. At the time of reinforcement, a small amount of food was squirted into the spoon through a hole at its bottom by a robotically pumped syringe under the electric control of a customized Arduino board that received digital commands from a computer.

The experiment was conducted in a dark acoustic chamber with infrared illumination. Throughout the entire training or testing session, the cat was monitored by an infrared webcam by the experimenter outside the chamber. The camera data acquired was analyzed in real time using an adapted region of interest–based motion detection algorithm using Computer Vision Toolbox (MathWorks) to identify specific cat behaviors that are crucial to the task, such as fixation and licking.

### Stimulus and procedures

A centered white dot on top of a constant gray background (0.3 cd/m^2^) was presented to the animal as a stimulus, as well as an orientation cue and a fixation cue, with the same diameter (32 pixel or 1.4°) but varying luminance. Stimulus luminance was calibrated with a photometer (Konica Minolta).

The orientation cue was a blinking white dot (50 cd/m^2^) switched on and off every 250 ms. Once the animal was engaged with the fixation dot, a test trial was started by an experimenter pressing a button. On the monitor, the orientation cue became a fixation cue, which was the exact same white dot except for being constantly displayed instead of blinking. At any time within this fixation phase, if the movement of the cat's head in a designated camera area exceeded a preset threshold, the trial was restarted by return of the orientation cue.

After a preset temporal period (1250, 2000, or 2750 ms) of successful fixation, a test stimulus was presented by introducing a brief luminance change to the white fixation dot. The duration and amplitude of the pulse of the luminance change were systematically manipulated as experiment variables, which will be referred to as pulse duration (PD) and pulse amplitude (PA).

**Figure 1.**
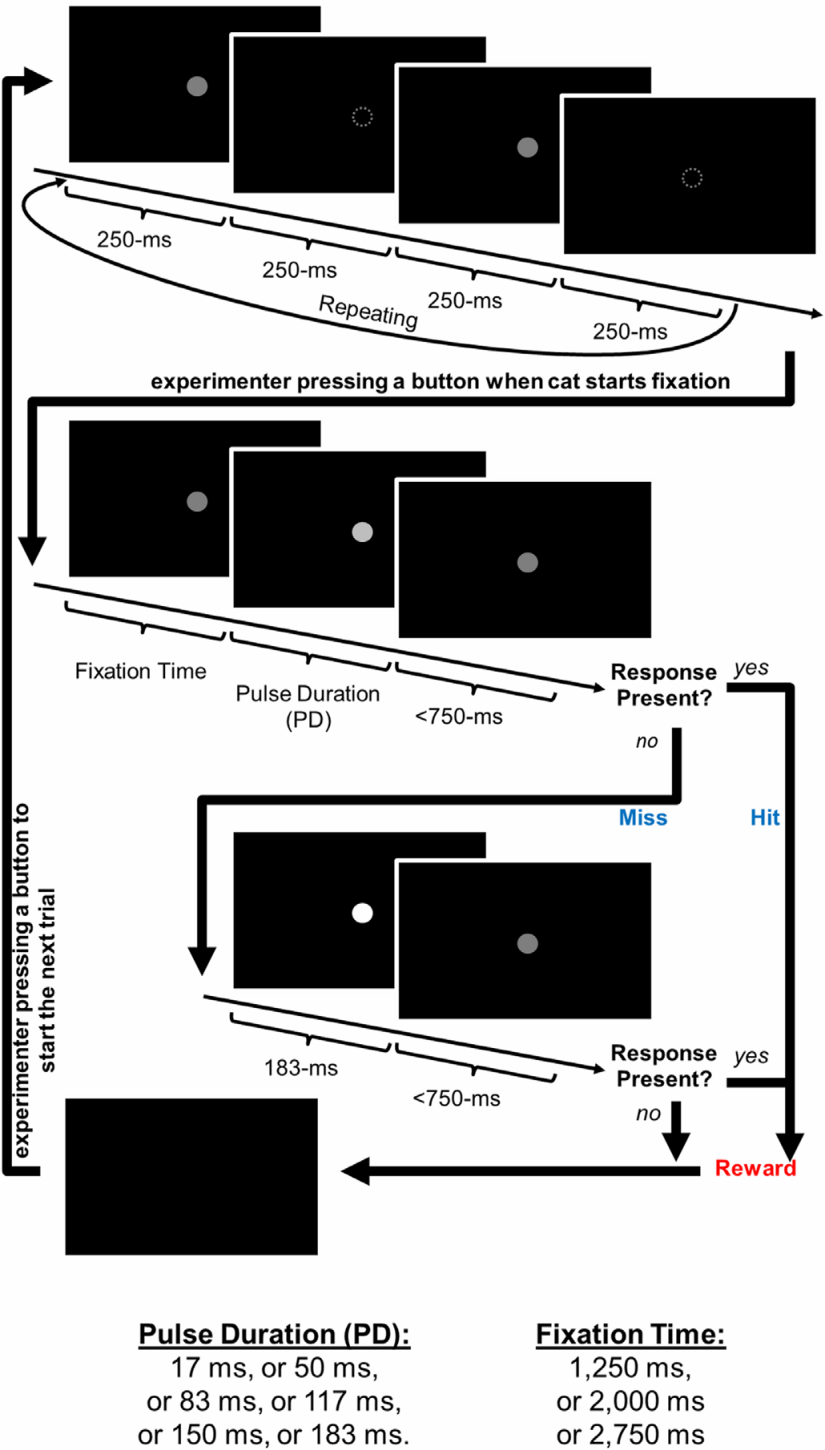
Schematic flow chart of a novel motivation-maintaining simple detection task.

After the onset of the testing stimulus, a 750-ms response window started to capture successful detection (i.e., cat unfixating and approaching the spoon) or failed detection (i.e., cat either fixating or unfixating without approaching the spoon). After a successful detection, the spoon would be filled immediately as a reinforcement at the end of the trial. After a failed detection, as long as the cat was still fixating (as in most cases), a second brief luminance change with the optimal PA and PD would be present, followed by a second 750-ms response window as well as the possible reinforcement, so as to strengthen the stimulus–response association and to maintain motivation (Figure 1).

### Experimental design

Cats were trained for 1 to 3 months before being able to perform the detection task at the optimal PA and PD with fixation time preset to 1250, 2000, 2750, or 3500 ms proficiently (success rate of >80%). Next, we determined a near-threshold PA for each cat by testing with varying PA between 0 and 70 cd/m^2^ (ref. to 50 cd/m^2^) while fixing the PD at 166 ms and looking at the psychometric function between success rate and PA.

Eventually, cats performed the detection task with PD randomly varying from 17 to 167 ms at the interval of 33 ms with the PA fixed at a threshold, subthreshold, or suprathreshold level. A test block consisted of 18 trials where each of six PDs were tested for three cycles with the same PA. The order of the tested PDs was randomized for each cycle. Each trial was designated with one of three fixation times (1250, 2000, or 2750 ms), in a way such that each PD was paired with each fixation time once and was used twice for one cycle. A typical testing session consisted of three testing blocks with three different PAs. The order of the tested PAs was counterbalanced across sessions. Each cat, except CTL, performed six to 18 testing sessions in total over 3 to 6 days.

### Data analysis

At the population level, we used a one-way repeated measure analysis of variance to determine the statistical significance of the difference between the means of success rate of detection for different stimulus durations. This was conducted within the Statistics and Machine Learning Toolbox (MathWorks) in MATLAB. At an individual level, we also applied nonlinear regressions to model the relationship between stimulus duration and success rate of detection using Curve Fitting Toolbox (MathWorks). Parameters for different template functions were approximated using a nonlinear least squares method. The Levenberg–Marquardt algorithm was used during the fitting procedure. Goodness of fit was converted to a z-score by Fisher transformation, and compared between linear and nonlinear exponential models using a Student's *t* test. The current data analysis did not include the trials when subjects failed to keep the fixation or made a response before stimulus onset, because the test stimulus was not present in these trials.

## Results

### Ability to detect a brief luminance deviant improved with PD

**Figure 2.**
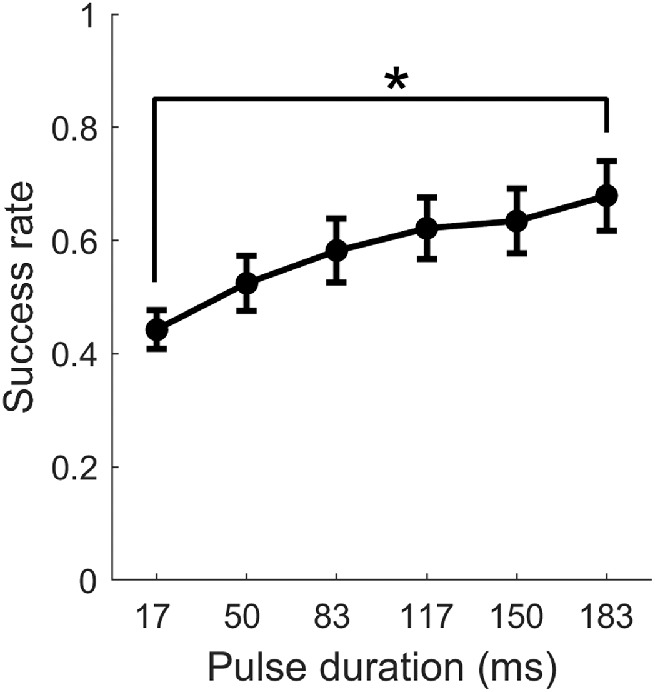
Grand average of success rate as a function of PD. Success rate of detecting pulses of luminance change with sub-, near-, or suprathreshold levels of PAs measured from five cats were averaged and plotted as a function of PD (*n* = 13). Error bar, standard error. *Significant difference shown by analysis of variance post hoc comparisons.

In total, we obtained 13 psychometric functions from five cats with sub-threshold, near-threshold, or suprathreshold PAs. On the averaged psychometric function, we found that 1) the rate of successful detection (i.e., success rate) increased monontonically with PD and 2) the slope of this psychometric function decreased with PD (Figure 2).

A one-way analysis of variance showed that the main effect of PD was statistically significant, *F*(5, 72) = 2.61, *p* = 0.032 < 0.05. This finding suggests that temporal integration does contribute to the detection of a weak visual stimulus. Next, we conducted *post-hoc* comparisons for all the possible combinations of two PDs. Significant difference was only observed between the shortest (17 ms) and the longest (183 ms) PD, *p* = 0.027 < 0.05.

To evaluate the effect of stimulus duration on the capacity of temporal integration, we calculated the increase in success rate of a 66-ms increase in stimulus duration, from 17 ms to 83 ms and from 117 ms to 183 ms, respectively. On average, the increase in success rate was larger for the short stimuli (approximately 14%) than the long stimuli (approximately 6%). Statistically, a paired two-sample Student's *t* test showed that the difference between the two increases in success rate was marginally significant, *p* = 0.058 > 0.05. Altogether, our data indicated that the visual system in cats can integrate stimulus input over time to help in the detection of a weak visual signal that is behaviorally relevant. However, as observed in the cat auditory system (Costalupes, 1983; Solecki & Gerken, 1990), cat visual temporal integration has a limited temporal capacity to benefit detection.

### Success rate saturated at long PDs

**Figure 3.**
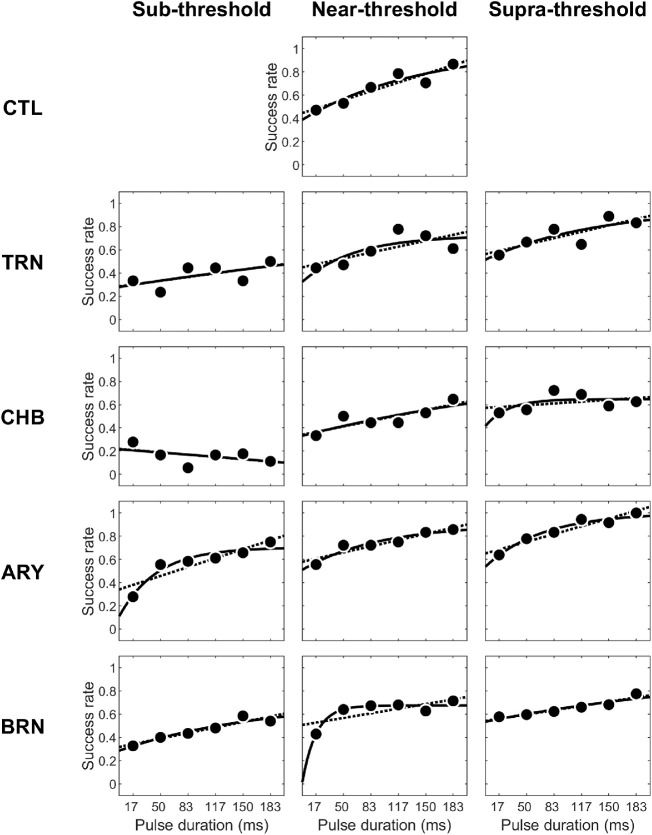
Individual psychometric functions. Thirteen individual psychometric functions, with each of five subjects shown in different rows and each of three PAs shown in different columns. In each plot, success rate was shown as a function of PD, with experimental data indicated by filled dots and linear or nonlinear best-fitting curve indicated by *dashed* or *solid line.*

To further characterize such limited temporal capacity, we individually fit each of the 13 psychometric functions with both a linear and a nonlinear customized model using *fit* function on MATLAB. The linear model used can be formulated as the following equation:

SR=a·PD+b

where *PD* represents for all PDs used in the experiment design and *SR* represents for success rate, respectively. Both *a* and *b* are free parameters and have no fitting bounds during the search of optimal fitness between the predicted and measured success rate. The nonlinear exponential model used can be formulated as the following equation:

$$SR=a\cdot e^{-b\cdot PD}+c$$

where *SR* and *PD* again represent success rate and PD acquired from the experiment, and *e* represents the base of the natural logarithm. To guide parameter searching, the upper bound of parameter *a* was set to 0. The bottom and upper bounds of parameter *c* were set to 0 and 1. The curves generated by the nonlinear model equation showed above using the best-fitting parameters for each of 13 testing sessions were plot and overlaid with experimentally measured success rates (Figure 3).

**Figure 4.**
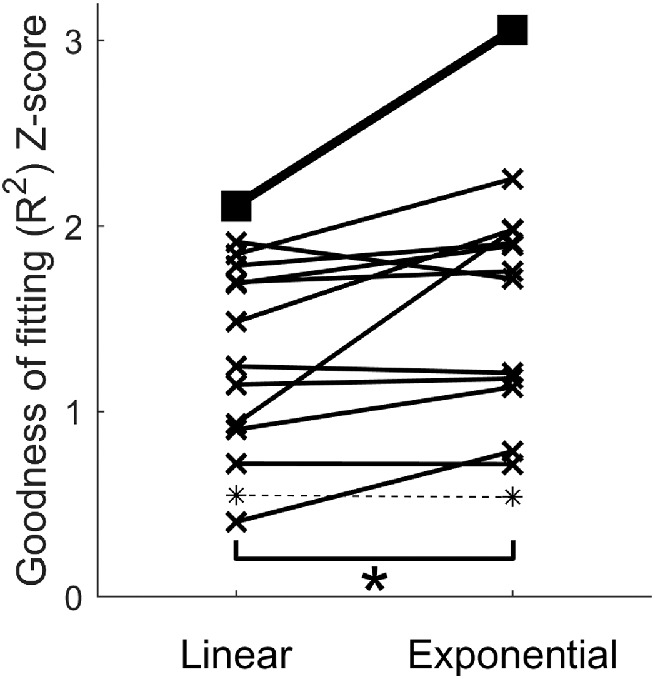
The comparison between linear and nonlinear curve fitting. For each individual data fitting process (*n* = 12), except for the apparent outlier (*), goodness of fitting (*r^2^*) was converted into Fisher's Z-score and compared between the linear model and the nonlinear exponential model, as indicated by (×). Statistical difference (*) between the two models were found (*p* < 0*.*05)*.* The averaged psychometric function was also fit with both models (■).

Using *R*^2^ as an index of goodness of fitting, we found that nine out of 13 psychometric functions were fit better with the nonlinear exponential model than with the linear model (Figure 4). Overall, Fisher *z*-transformation of *R*^2^ for the nonlinear exponential model was significantly higher than that for the linear model, *p* = 0.036 < 0.05.

These results suggest that the nonlinear exponential model can be an authentic and useful simplification of the psychometric relationship between successful detection and stimulus duration. From the parameters derived from the fitting of the averaged data, it can be estimated that the benefit of temporal integration for detecting a visual stimulus decays to 36.8% with each 101.4 ms increase of integration period.

As an attempt to explore the possibility of other nonlinear models in fitting our experimental results, we also tried a sigmoid function and semilogarithmic piecewise linear model as an alternative to an exponential function (Supplementary Figure S1). However, with our current dataset, these two models did not seem to demonstrate any benefit in goodness of fitting when compared with linear and exponential models (Supplementary Figure S2).

### Saturated success rate continued to increase with PA

**Figure 5.**
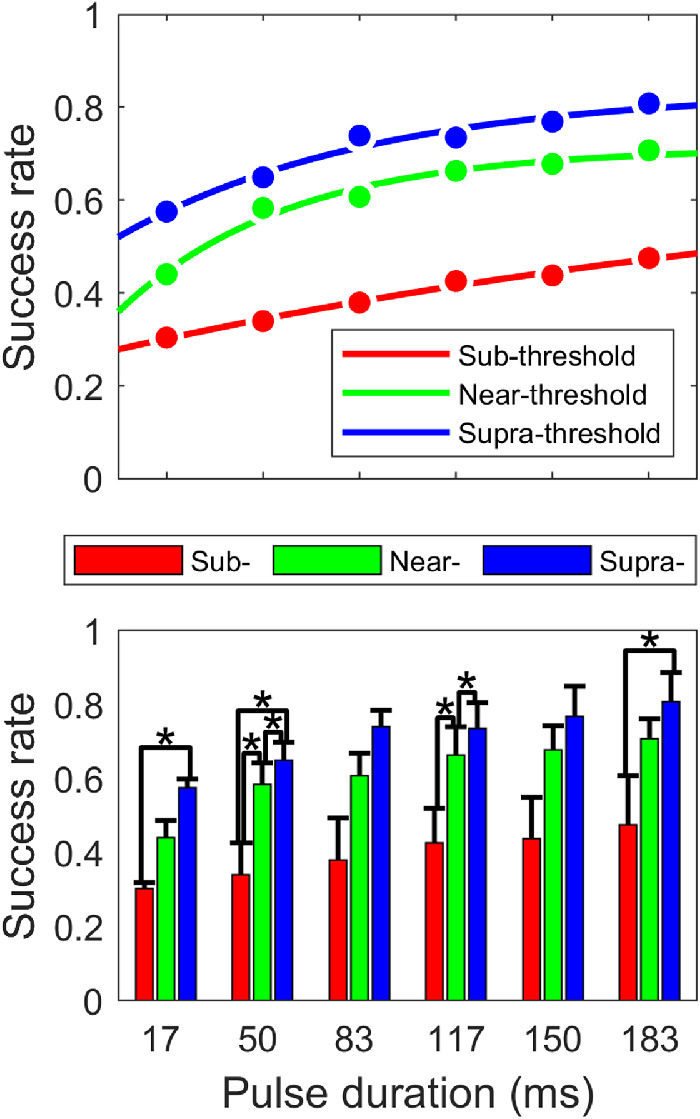
Effect of stimulus intensity on psychometric functions. *Top*, averaged psychometric functions for each of sub-, near-, suprathreshold PAs were plot in *red*, *green* and *blue*, respectively. Bottom, bars with different colors (same as in top) compared averaged success rate for each of six PDs (*n* = 4). Error bar, standard error. *p < 0.05 in analysis of variance multiple comparison test.

To ensure that the saturation of the success rate was not a ceiling effect limited by cats’ proficiency in performing the task in general, in four of the five cats, three different PAs were used. Therefore, we were also able to examine the effect of PA on success rate for each PD. Overall, three psychometric functions stacked up in the order of corresponding PA without crossing each other (Figure 5, top). A two-way (3 × 6) repeated measured analysis of variance showed significant effects of both PD, *F*(5, 15) = 11.07, *p* < 0.001, and PA, *F*(2, 6) = 30.74, *p* < 0.001. Although no significant interaction was found, we applied the test of simple effect of PA at each level of PD (Figure 5, bottom). The effect of PA suggested that the task proficiency is not likely the reason for the saturated performance.

We noticed that the trend we observed from population average was more consistently present for near- and suprathreshold than subthreshold PA (luminance) levels. Deaf subjects seemed to show more consistent individual psychometric functions than hearing subjects, although we can't make any conclusion from our limited number of subjects.

### Summary

The data from this study showed that successful detection of a visual stimulus was modulated by the stimulus duration in the range between approximately 20 and 200 ms. At a population level, the effect of increasing stimulus duration on task performance was more prominent for short stimuli than long stimuli. For each individual testing session, the psychometric function was often better fit by a nonlinear exponential model than a linear model. The duration effect and the unique shape of the psychometric functions persist with different stimulus intensities.

## Discussion

### Leaky integrator and energy integrator

Duysens (1991) used the terms “leaky integrator” and “energy integrator” to describe two of the most important aspects of temporal integration in area 17 neurons, which could also apply to behavioral measurements. A “leaky integrator” describes an input–output relationship where the input is added together over a finite-length time window. With intensity constant over time, prolonging the duration of the input (i.e., stimulus) increases the output (i.e., perception) before saturation is reached, which defines the critical duration. An “energy integrator,” in contrast, takes both intensity and time as indirect factors of the output. Instead, the product of intensity and time, energy, is the direct factor. This term emphasizes the interchangeability of intensity and time in temporal integration (Duysens et al., 1991).

In human subjects, many previous studies have characterized visual temporal integration by measuring the intensity (luminance or contrast) thresholds as a function of stimulus duration (Gorea & Tyler, 1986; Rashbass, 1970) or the intensity level at each stimulus duration that is perceptually equivalent to a standard stimulus with both parameters fixed (Aiba & Stevens, 1964; Rieiro et al., 2012; Stévens & Hall, 1966). Both approaches allow for the observation of an energy integrator as an iso-brightness curve, where any stimulus featuring an intensity–duration pair on the curve is equally detectable or equally bright. Although these studies have provided valuable insights in understanding visual temporal integration, there are some shortcomings as well. First, compared with estimating response rates on average, it takes many more trials to measure stimulus thresholds. Second, it is difficult to avoid rise–decay slope covarying with stimulus intensity when using a computer monitor to deliver the stimulus. Thus, given the goal of this study, it is sufficient, and probably more straightforward, to use a stimulus with fixed intensity levels and measuring success rate of detection.

### Bloch's law versus broca-sulzer's law

Although almost all relevant studies reported a generally monotonic increase of perceived brightness with increasing stimulus duration as in Bloch's law (Bloch, 1885; Harwerth, Boltz, & Smith, 1980), a handful of studies have also reported a small negative marginal effect of increasing stimulus duration for medium–long duration stimuli (Breitmeyer & Ganz, 1977; Broca & Sulzer, 1902; Kelly & Savoie, 1978; Rieiro et al., 2012; Roufs, 1974). This finding was first documented and named in 1902 (Broca & Sulzer, 1902). Recently, the underlying mechanism for the difference observed in Broca–Sulzer's law compared with Bloch's law was debated (Gorea & Tyler, 2013). An earlier study proposed that Broca–Sulzer's law could be the result of inhibition, by showing that reducing the spatial frequency of a grating stimulus could effectively modify the shape of the psychometric function observed in Bloch's law to that of Broca–Sulzer's law (Gorea & Tyler, 1986). The simulation in this study showed that the extra dips observed in Broca–Sulzer's law arises from a second inhibitory phase following excitation in the impulse–response function (the Fourier transform of a band-passing temporal modulation transfer function), which is a characteristic for grating stimuli of spatial frequency lower than 3-cyc/deg but not higher (Robson, 1966). However, a more recent study (Rieiro et al., 2012) proposed that subject bias is the reason for observing Bloch's law rather than Broca–Sulzer's law. They observed, in the same group of participants, Bloch's law with a blocked design and Broca–Sulzer's law with au unblocked design. Rieiro et al. (2012) therefore implied that Broca–Sulzer's law is a fundamental feature of visual system whereas Bloch's law demonstrates a perceptual representation of sensory input bias as in “brightness constancy” (Gilchrist et al., 1999).

In the present experimental design, the same luminance paired with different stimulus durations was used for each testing session. This design was similar to the blocked design used in an earlier experiment where Broca–Sulzer's law was observed (Rieiro et al., 2012). However, the shape of the averaged psychometric function from our dataset was closer to Bloch's law. Because individual psychometric functions tend to be noisy, it is difficult to determine if they favor either law. Also, we do not know if subject bias observed in human subjects would be comparable in the cat visual system. In a future study, it would be interesting to compare our results from cats with those from human subjects.

### Neural correlations of temporal integration in sensory systems

It is also important to note that such an intensity–duration trade-off is not unique to the visual system. For instance, Stévens and Hall (1966) showed that both visual brightness and auditory loudness grew with stimulus duration and eventually reached saturation (Stévens & Hall, 1966). Similarly, for tactile perception, overestimation of speed of whisker vibration occurring by simply increasing stimulus duration has been identified in both rats (Fassihi, Akrami, Pulecchi, Schönfelder, & Diamond, 2017) and monkeys (Luna, Hernández, Brody, & Romo, 2005). Fassihi et al. (2017) showed that neuronal activities after stimulus offset in vM1 (vibrissal motor cortex) but not vS1 (vibrissal sensory cortex) is modulated by both the speed and duration of tactile vibration. In this study, tactile stimuli with longer durations led to higher speed of vibration perceived by rats, and higher firing rates recorded from vM1 neurons. This finding suggests that temporal integration of sensory input may also be processed outside of primary sensory cortices. However, in this particular case, the activity of vM1 neurons may be highly task specific or involved in the motor planning in response to sensory experience, rather than the sensory experience itself. Although the temporal integration of sensory input is shown to be largely accounted for by the integrative properties of sensory receptors, as well as sensory neurons in each individual sensory modality, it has been speculated that cortical processing may play a role at longer timescales (Mongillo & Loewenstein, 2017).

Several potential neural mechanisms of visual temporal integration have been proposed based on electrophysiological investigations. Typically, in vivo extracellular recording has been performed along the visual pathway to record neuronal firing in response to visual stimuli of varying duration. Examining the maximum neuronal firing rate, Duysens et al. (1991) showed that neuronal activity in cat area 17 were modulated by stimulus durations up to 80 ms, which is 50% to 100% higher than those previously reported in retinal ganglion cells (Levick & Zacks, 1970; Scheich & Korn, 1971). Duysens et al. (1991) was the first study to highlight the role of visual cortex in temporal integration, where the time scale is very close to that found in behavioral studies. Another way of investigating temporal integration is to present a fixed-length stimulus (e.g., 500 ms), but iteratively analyze neural activities in a varying length window aligned to the stimulus onset, if we can assume the effect of stimulus after the end of response window is negligible (but see Enns & Di Lollo, 2000). Neurometric functions constructed using this method for neurons in monkey primary visual cortex and medial temporal area showed that the marginal benefit of prolonging the response window on neuron's capacity of orientation discrimination decreased over time (Goris et al., 2018).

## Auditory and visual temporal integration

Auditory stimuli, such as pure tones and noise, have been used in studies of temporal integration in many animal models (see Heil et al., 2017, for a review), including cats (Costalupes, 1983; Solecki & Gerken, 1990). In cats, the detection thresholds of sound levels for pure tones were measured for stimulus durations ranging from 50 to 1000 ms. The sound level thresholds were found to decay with stimulus duration; with decay constants ranging between 100 and 1000 ms that were inversely correlated with pure tone frequency (Costalupes, 1983). Unfortunately, no comparable study investigating visual temporal integration using the same animal model could be identified. One of the obstacles that discourages such a study is the difficulty of engaging a cat in fixation that is sufficiently stable over the entire period of stimulus delivery.

In this study, we implemented a training paradigm that can quantify animal behavior with online image processing, and thus successfully trained five cats to perform a simple fixation detection task. In this task, the cats were only loosely restrained in a canvas bag with a large freedom of movement in their head positions. Taking advantage of this training paradigm, we were able to reliably measure a cat's ability to detect brief pulses of luminance change in the fixation dot. By constructing the psychometric functions of varying PD, we were able to quantify the capacity for visual temporal integration in cat. For future studies, this task can be easily adapted into a bimodal version, in which case a direct comparison between auditory and visual temporal integration can be made in the same subject.

## Conclusions

Considering the behavioral and electrophysiological findings in the current and previous studies, we believe that the cat can be a promising model for future research to answer unresolved issues in visual temporal integration.

## Supplementary Material

Supplement 1
