# Supplementary material for: The limited capacity of visual temporal integration in cats
Source: J Vis. 2020 Aug 27;20(8):28. doi: 10.1167/jov.20.8.28 (PMC7453054; doi:10.1167/jov.20.8.28)
Supplement: Supplement 1 [file jovi-20-8-28_s001.pdf]

Supplementary Results

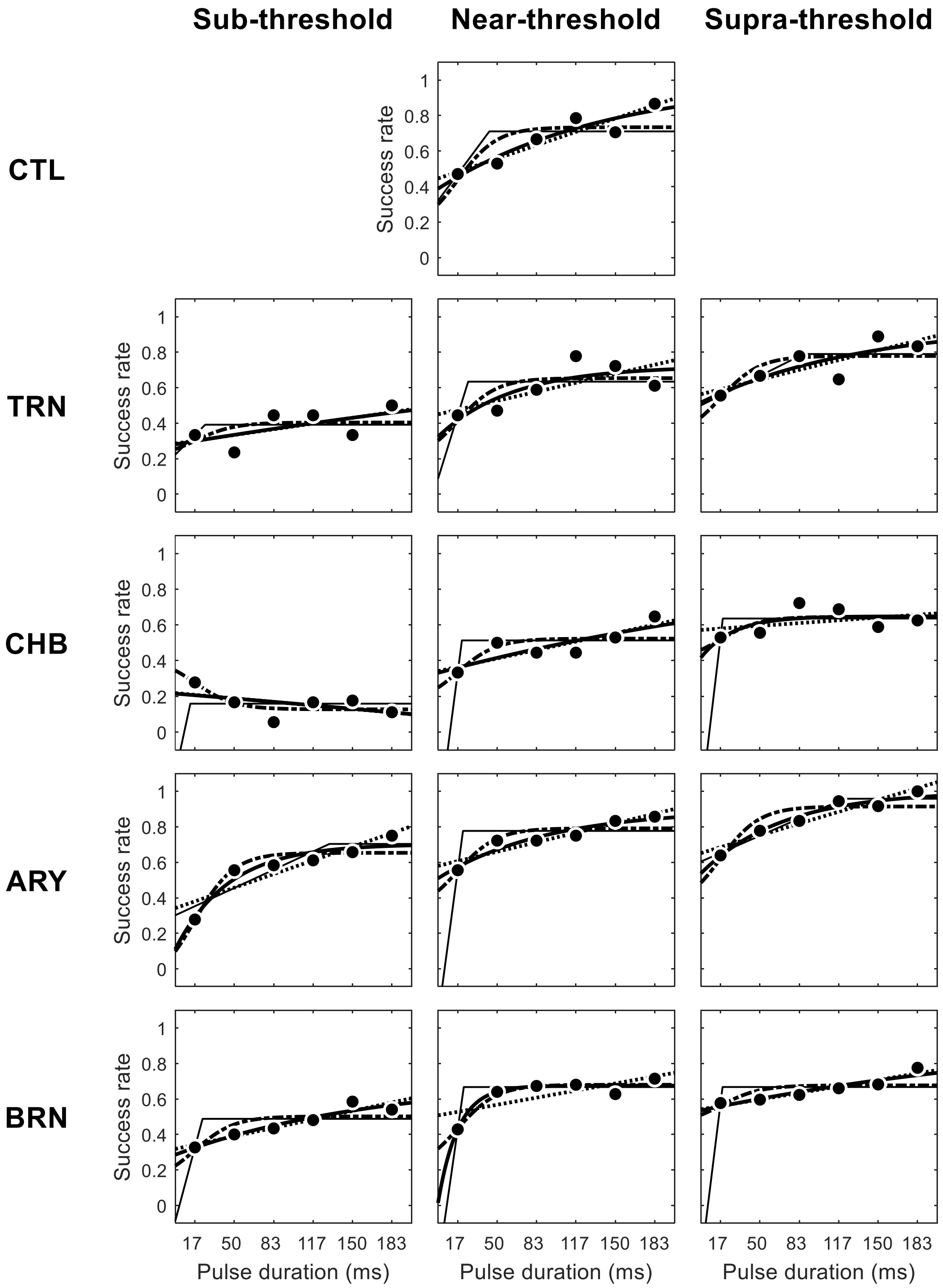

Figure 1S. Individual psychometric functions fitting with linear and multiple non-linear models. As a supplementary result to Figure 3, three different non-linear models (thick solid lines, exponential function; dashdotted lines, sigmoid function; a piecewise function, thin

solid lines) were used to fit success rate observed from our psychophysics experiment, as well the linear mode (dotted lines). To improve the stability in the fitting results, each fitting was repeated for 10 times and the one resulting the highest goodness of fitting was present here and kept for further analysis.

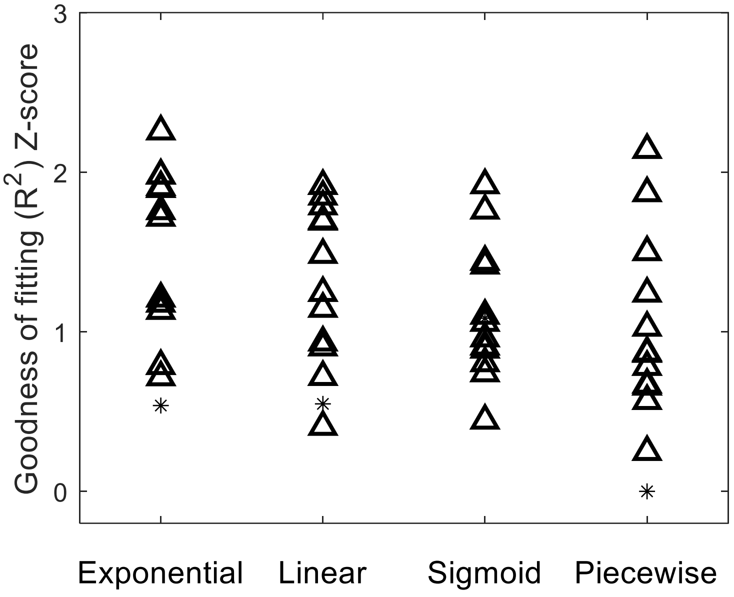

Figure 2S. The comparison of goodness of fitting between linear and multiple non-linear models. As a supplementary result to Figure 4, goodness of fitting (r^2) derived from three different non-linear models as well as the linear model was converted into Fisher's Z-score and present here. One-way ANOVA showed no significant main effect of modeling method on goodness of fitting.
